# A nickel(II) complex with an unsymmetrical tetra­dentate chelating ligand derived from pyridine-2,6-dicarbaldehyde and 2-amino­thio­phenol

**DOI:** 10.1107/S2056989023006692

**Published:** 2023-08-04

**Authors:** Firas Khalil Al-Zeidaneen, Christopher E. Anson, Annie K. Powell

**Affiliations:** a Department of Chemistry and Chemical Technology, Tafila Technical University, Tafila, Jordan; b Karlsruher Institut für Technologie (KIT), Institut für Anorganische Chemie, Engesserstrasse 15, 76131 Karlsruhe, Germany; c Karlsruher Institut für Technologie (KIT), Institut für Nanotechologie, Hermann von-Helmholtz Platz 1, 76344 Eggenstein-Leopoldshafen, Germany; University of Kentucky, USA

**Keywords:** crystal structure, nickel, chelate ligand, ligand modification

## Abstract

[(2-{[6-(1,3-Benzo­thia­zol-2-yl)pyridin-2-yl]carbonyl­aza­nid­yl}phen­yl)sulf­anido]nickel(II), [Ni(C_19_H_11_N_3_OS_2_)], crystallizes in the centrosymmetric monoclinic space group *P*2_1_/*n* with one mol­ecule in the asymmetric unit. The expected ligand, a bis-Schiff base derived from pyridine-2,6-dicarbaldehyde and 2-amino­thio­phenol, had modified *in situ* in a both unexpected and unsymmetrical fashion. One arm had cyclized to form a benzo[*d*]thia­zol-2-yl functionality, while the imine linkage of the second arm had oxidized to an amide group. The geometry about the central Ni^II^ atom is distorted square-planar N_3_S. The mol­ecules form supra­molecular face-to-face dimers *via* rather strong π–π stacking inter­actions, with these dimers then linked into chains *via* pairwise C—H⋯O inter­actions.

## Abstract 2

[(2-{[6-(1,3-Benzo­thia­zol-2-yl)pyridin-2-yl]carbonyl­aza­nid­yl}phen­yl)sulfanido]nickel(II) crystallizes in the centrosymmetric monoclinic space group *P*2_1_/*n*. Both arms of the expected bis-Schiff base ligand based on pyridine-2,6-dicarbaldehyde and 2-amino­thio­phenol had oxidized; one by cyclization to a benzo­thia­zole, the other by oxidation of its imine linkage to the corresponding amide.

## 1. Chemical context

In recent decades, Schiff base chemistry has proved a both fruitful and flexible source of organic ligands for coordination chemistry. Double Schiff bases, derived from two equivalents of an amine with a pyridine-2,6-dicarbaldehyde or a 2,6-phenoldicarbaldehyde, provide planar multidentate ligands that can mimic the properties of macrocyclic ligands without themselves being strictly cyclic. 
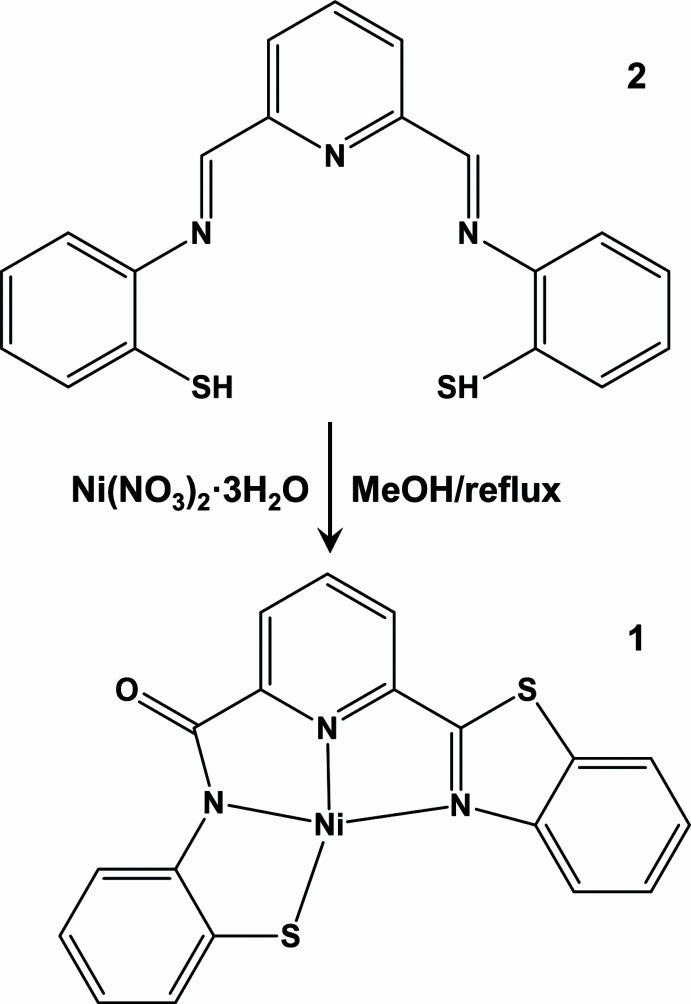


In this context, we were inter­ested in developing such double Schiff base ligands that are redox-active, and targeted ligand **2**, with the intention that formation or cleavage of a di­sulfide bond would give the necessary redox activity. However, *in situ* formation of the ligand through condensation of pyridine-2,6-dicarbaldehyde with two equivalents of 2-amino­thio­phenol, followed by reaction with Ni(NO_3_)_2_·3H_2_O in refluxing methanol, did not yield the expected Ni^II^ complex of ligand **2**, but instead gave the title complex **1** in good yield, in which the two ligand arms have both been oxidized, but in very different manners.

## 2. Structural commentary

**Figure 1:**
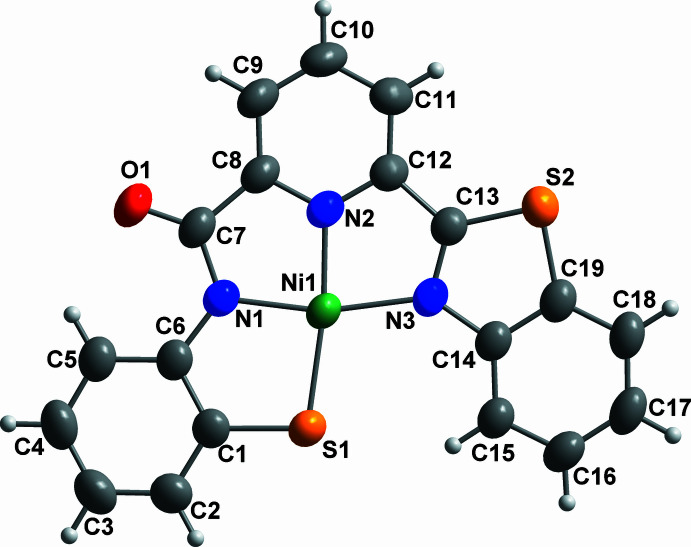
Mol­ecular structure of **1** with atom labelling; displacement ellipsoids represent 50% probability levels

Compound **1** crystallizes in the monoclinic space group *P*2_1_/*n* with one mol­ecule in the asymmetric unit (Fig. 1 ▸). Selected bond lengths and angles are listed in Table 1 ▸. The central Ni1 atom has a distorted square-planar geometry with an N_3_S donor set, in which the *X*—Ni1—*Y* angles (Table 1 ▸) differ by up to 15° from either 90° or 180°. The whole mol­ecule can be considered as planar, with the r.m.s. deviation of the atoms from their mean plane being 0.0867 Å, and the oxygen atom O1 showing the largest deviation from the plane of 0.210 (3) Å. It is immediately clear from the structure that the expected nickel complex of ligand **2** had not formed. Instead, the two ligand arms have each been differently oxidized in such a way as to yield a very unsymmetrical ligand.

The expected imine linkage of arm **1** (that including atom S1) has been oxidized to an amido functionality, as is clear from the short C7—O1 bond length of 1.229 (4) Å. There are two possible scenarios here. One is that one of the aldehyde groups oxidized to the corresponding carb­oxy­lic acid, followed by reaction with the amino­thio­phenol to form the amide. The other is that the Schiff base arm formed as expected, but with subsequent nucleophilic attack by water on the imino carbon atom, followed by oxidation to yield the amide. No significant electron density corresponding to a possible H atom could be found near S1, so this can be assigned as a deprotonated thio­pheno­lato group. C15 forms an intra­molecular C—H⋯S hydrogen bond to S1 (Table 2 ▸), while any H atom bonded to S1 would lead to an unrealistic short contact to H15. Ni^II^ complexes of ligands containing such amido­benzene­thiol­ate units have previously been reported (Seratne *et al.*, 2018 ▸), and their Ni—N and Ni—S distances [1.874 (3)–1.896 (9) Å and 2.126 (4)–2.1343 (9) Å] are similar to the corresponding bond lengths in **1**, 1.871 (2) and 2.1508 (9) Å, respectively, although Ni1—N1 in **1** is slightly shorter, and Ni1—S1 slightly longer, than in these literature values.

The other arm of the ligand is also oxidized relative to the expected structure of **2**, but here this has involved an oxidative cyclization, in which the sulfur atom S2 has initially attacked the imine carbon C13 to give a benzo[*d*]thia­zol-2-yl functional group. Such oxidative cyclization has been previously observed in a related ligand system in which a 2,6-phenoldicarbaldehyde was condensed with two equivalents of 2-amino­thio­phenol (Gulcan *et al.*, 2014 ▸). An Ni^II^ complex with a chelating 2-(2′-pyrid­yl)-benzo­thia­zole ligand has previously been structurally characterized (Patel *et al.*, 2010 ▸), in which the Ni—N(thia­zole) distance was 2.116 (2) Å, thus significantly longer than Ni1—N3 in **1** [1.952 (2) Å]. However, the reported complex was octa­hedral rather than square planar, and the benzo­thia­zole N atom was *trans* to an aqua ligand rather than the negatively charged deprotonated N atom in **1**. The benzo­thia­zolyl arm is clearly neutral, while the other formally carries negative charges on S1 and N1, with the ligand as a whole thus a dianion. This is consistent with the calculated valency for Ni1 of 2.14 obtained from bond-valence-sum analysis (Brese & O’Keeffe, 1991 ▸; Liu & Thorp, 1993 ▸).

## 3. Supra­molecular features

**Figure 2:**
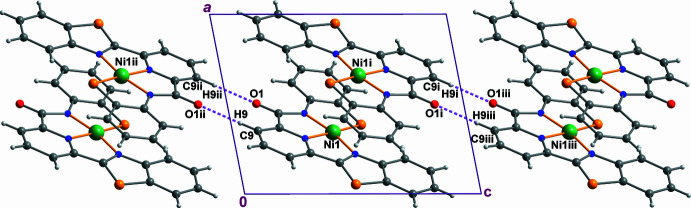
Supra­molecular inter­actions in the crystal structure of **1**. Hydrogen bonds are shown as purple dashed lines. Symmetry codes: (i) −*x* + 1, −*y* + 1, −*z* + 1; (ii) −*x* + 1, −*y* + 1, −*z*; (iii) *x*, *y*, *z* + 1.

In the crystal, the mol­ecules of **1** are organized into centrosymmetric π-stacked supra­molecular dimers (Fig. 2 ▸), with the shortest inter­molecular distance within such a dimer involving the two respective nickel atoms, with Ni1⋯Ni1^i^ = 3.3305 (9) Å [symmetry code: (i) −*x* + 1, −*y* + 1, −*z* + 1]. These dimers are then linked into chains running parallel to the crystal *c*-axis by pairwise C9—H9··O1^ii^ H-bonds (Table 2 ▸) [symmetry code: (ii) −*x* + 1, −*y* + 1, −z].

## 4. Database survey

A survey of the Cambridge Structural Database (CSD, v5.44, including updates to June 2023; Groom *et al.* 2016 ▸) showed that no crystal structure of **1**, nor any other complex of the same or related unsymmetrical ligand, nor the free ligand itself, has previously been reported. Two complexes of the bis-deprotonated target ligand **2** have been reported: the Zn^2+^ complex BTAQZN10 (Goedken & Christoph, 1973 ▸) and the methyl­thallium complex TPAMTL (Henrick *et al.*, 1977 ▸). In a further 13 structures, the two S atoms are bonded to an organic functional group (usually methyl, but in some cases the sulfur atoms are linked *via* di- or tri­methyl­ene chains to form a macrocycle); in these ligands the S atoms are unable to carry a negative charge. The structures of six complexes of the symmetrical ligand 2,6-*bis*-(benzo[d]thia­zol-2-yl)pyridine were found, but all with metals other than nickel. 11 structures were found for complexes with ligands in which a pyridine ring carried either one or two doubly deprotonated 2-thio­pheno­lato­amido groups, but again no nickel complexes were among these. The structures of 15 further complexes, in which the S atom(s) of these ligands carry an organic functional group, were found. Six of these were nickel complexes, but were all octa­hedral hexa­coordinate, in contrast to the square-planar **1**.

## 5. Synthesis and crystallization

2-Amino­thio­phenol (63 mg, 0.50 mmol) in methanol (5 ml) was added to a solution of pyridine-2,6-dicarbaldehyde (34 mg, 0.25mmol) in methanol (15ml). The mixture was stirred for 15 minutes at room temperature before Ni(NO_3_)_2_·3H_2_O (60 mg, 0.25 mmol) was added as a solid. The mixture was heated under reflux for 2 h, after which it was allowed to cool to room temperature, was filtered, and the filtrate left to stand undisturbed. Black needle-shaped crystals of the compound, suitable for X-ray diffraction, were obtained as the methanol evaporated slowly after three days. The resulting crystals were filtered and washed with cold methanol. Yield (35%) based on Ni.

Elemental analysis calculatedd (%) for C_19_H_11_N_3_NiOS_2_: C 54.28, H 2.62, N 10.00; found: C 54.16, H 2.57, N 9.91

IR: *ν* (cm^−1^): 3282 (*w*), 3263 (*w*), 3238 (*w*), 3224 (*m*), 3207 (*w*), 1640 (*s*), 1521 (*s*), 1446 (*s*), 1392 (*w*), 1369 (*m*), 1324 (*w*), 1221 (*w*), 1190 (*w*), 1169 (*w*), 1129 (*m*), 1067 (*m*), 870 (*w*), 837 (*m*), 795 (*s*), 771 (*w*), 755 (*w*), 710 (*w*), 620 (*w*), 559 (*w*), 507 (*w*), 480 (*w*), 461 (*w*), 438 (*w*), 423 (*w*).

## 6. Refinement

Crystal data, data collection and structure refinement details are summarized in Table 3 ▸. Non-H atoms were refined anisotropically. H atoms were placed in geometrically idealized positions, riding on their respective C atoms with *U*_iso_(H) = 1.2*U*_eq_(C)

## Supplementary Material

Crystal structure: contains datablock(s) I. DOI: 10.1107/S2056989023006692/pk2692sup1.cif

Structure factors: contains datablock(s) I. DOI: 10.1107/S2056989023006692/pk2692Isup2.hkl

Supporting information file. DOI: 10.1107/S2056989023006692/pk2692Isup3.cdx

CCDC reference: 2285993

Additional supporting information:  crystallographic information; 3D view; checkCIF report

## Figures and Tables

**Table 1:** Selected geometric parameters (Å, °)

Ni1—S1	2.1508 (9)	N1—C6	1.405 (4)
Ni1—N1	1.871 (2)	N1—C7	1.354 (4)
Ni1—N2	1.843 (2)	N2—C8	1.343 (3)
Ni1—N3	1.952 (2)	N2—C12	1.332 (4)
S1—C1	1.761 (3)	N3—C13	1.328 (4)
S2—C13	1.706 (3)	N3—C14	1.395 (3)
S2—C19	1.733 (3)	Ni1—Ni1^i^	3.3305 (9)
O1—C7	1.229 (4)		
			
N1—Ni1—S1	89.40 (8)	C1—S1—Ni1	97.34 (10)
N1—Ni1—N3	165.47 (10)	C13—S2—C19	88.68 (14)
N2—Ni1—S1	172.45 (8)	O1—C7—N1	128.0 (3)
N2—Ni1—N1	83.74 (11)	O1—C7—C8	120.7 (3)
N2—Ni1—N3	81.75 (10)	N1—C7—C8	111.2 (2)
N3—Ni1—S1	105.05 (8)		

Symmetry code: (i) 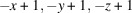 .

**Table 2:** Hydrogen-bond geometry (Å, °)

*D*—H⋯*A*	*D*—H	H⋯*A*	*D*⋯*A*	*D*—H⋯*A*
C9—H9⋯O1^ii^	0.93	2.27	3.135 (4)	155
C15—H15⋯S1	0.93	2.66	3.420 (3)	139

Symmetry code: (ii) 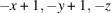 .

**Table 3:** Experimental details

Crystal data	
Chemical formula	[Ni(C_19_H_11_N_3_OS_2_)]
*M*_r_	420.14
Crystal system, space group	Monoclinic, *P*2_1_/*n*
Temperature (K)	291
*a*, *b*, *c* (Å)	8.6790 (2), 17.3282 (7), 11.2211 (4)
β (°)	101.002 (3)
*V* (Å^3^)	1656.54 (10)
*Z*	4
Radiation type	Cu *K*α
μ (mm^−1^)	4.16
Crystal size (mm)	0.43 × 0.04 × 0.03

Data collection	
Diffractometer	SuperNova, Dual, Cu at zero, Eos
Absorption correction	Multi-scan (*CrysAlis PRO*; Rigaku OD, 2018 ▸)
*T*_min_, *T*_max_	0.675, 1.000
No. of measured, independent and observed [*I* > 2σ(*I*)] reflections	9403, 3155, 2567
*R*_int_	0.022
(sin θ/λ)_max_ (Å^−1^)	0.613

Refinement	
*R*[*F* ^2^ > 2σ(*F* ^2^)], *wR*(*F* ^2^), *S*	0.039, 0.118, 1.04
No. of reflections	3155
No. of parameters	235
H-atom treatment	H-atom parameters constrained
Δρ_max_, Δρ_min_ (e Å^−3^)	0.44, −0.36

Computer programs: *CrysAlis PRO* (Rigaku OD, 2018 ▸), *SHELXT2018/2* (Sheldrick, 2015*a* ▸), *SHELXL2018/3* (Sheldrick, 2015*b* ▸), *DIAMOND* (Brandenburg, 2017 ▸) and *OLEX2* (Dolomanov *et al.*, 2009 ▸).

## supplementary crystallographic information

### Crystal data

**Table:**

[Ni(C_19_H_11_N_3_OS_2_)]	*F*(000) = 856
*M**_r_* = 420.14	*D*_x_ = 1.685 Mg m^−^^3^
Monoclinic, *P*2_1_/*n*	Cu *K*α radiation, λ = 1.54184 Å
*a* = 8.6790 (2) Å	Cell parameters from 3558 reflections
*b* = 17.3282 (7) Å	θ = 2.5–69.9°
*c* = 11.2211 (4) Å	µ = 4.16 mm^−^^1^
β = 101.002 (3)°	*T* = 291 K
*V* = 1656.54 (10) Å^3^	Needle, black
*Z* = 4	0.43 × 0.04 × 0.03 mm

### Data collection

**Table:**

SuperNova, Dual, Cu at zero, Eos diffractometer	3155 independent reflections
Radiation source: micro-focus sealed X-ray tube, SuperNova (Cu) X-ray Source	2567 reflections with *I* > 2σ(*I*)
Mirror monochromator	*R*_int_ = 0.022
Detector resolution: 8.0534 pixels mm^-1^	θ_max_ = 71.1°, θ_min_ = 4.8°
ω scans	*h* = −7→10
Absorption correction: multi-scan (CrysAlisPro; Rigaku OD, 2018)	*k* = −20→21
*T*_min_ = 0.675, *T*_max_ = 1.000	*l* = −13→13
9403 measured reflections	

### Refinement

**Table:**

Refinement on *F*^2^	Primary atom site location: dual
Least-squares matrix: full	Secondary atom site location: difference Fourier map
*R*[*F*^2^ > 2σ(*F*^2^)] = 0.039	Hydrogen site location: inferred from neighbouring sites
*wR*(*F*^2^) = 0.118	H-atom parameters constrained
*S* = 1.04	*w* = 1/[σ^2^(*F*_o_^2^) + (0.0625*P*)^2^ + 0.6572*P*] where *P* = (*F*_o_^2^ + 2*F*_c_^2^)/3
3155 reflections	(Δ/σ)_max_ < 0.001
235 parameters	Δρ_max_ = 0.44 e Å^−^^3^
0 restraints	Δρ_min_ = −0.36 e Å^−^^3^

### Special details

**Table:**

Geometry. All esds (except the esd in the dihedral angle between two l.s. planes) are estimated using the full covariance matrix. The cell esds are taken into account individually in the estimation of esds in distances, angles and torsion angles; correlations between esds in cell parameters are only used when they are defined by crystal symmetry. An approximate (isotropic) treatment of cell esds is used for estimating esds involving l.s. planes.

### Fractional atomic coordinates and isotropic or equivalent isotropic displacement parameters (Å^2^)

**Table:**

	*x*	*y*	*z*	*U*_iso_*/*U*_eq_	
Ni1	0.34262 (5)	0.45461 (3)	0.42355 (4)	0.04539 (17)	
S1	0.38510 (9)	0.35936 (5)	0.54841 (7)	0.0545 (2)	
S2	0.05248 (9)	0.65284 (5)	0.47762 (7)	0.0564 (2)	
O1	0.5026 (3)	0.41006 (16)	0.1239 (2)	0.0731 (7)	
N1	0.4482 (3)	0.40139 (14)	0.3182 (2)	0.0475 (5)	
N2	0.3088 (3)	0.52692 (15)	0.3009 (2)	0.0479 (5)	
N3	0.2213 (2)	0.52852 (15)	0.4992 (2)	0.0464 (5)	
C1	0.5054 (3)	0.30515 (17)	0.4699 (3)	0.0510 (7)	
C2	0.5821 (4)	0.23886 (19)	0.5187 (3)	0.0613 (8)	
H2	0.569661	0.221941	0.594992	0.074*	
C3	0.6770 (4)	0.1975 (2)	0.4553 (4)	0.0691 (9)	
H3	0.729053	0.153451	0.489113	0.083*	
C4	0.6935 (4)	0.2223 (2)	0.3413 (4)	0.0679 (9)	
H4	0.756201	0.194189	0.298354	0.082*	
C5	0.6186 (3)	0.28795 (19)	0.2902 (3)	0.0594 (8)	
H5	0.630444	0.303622	0.213204	0.071*	
C6	0.5241 (3)	0.33128 (17)	0.3547 (3)	0.0486 (6)	
C7	0.4485 (3)	0.43536 (19)	0.2096 (3)	0.0531 (7)	
C8	0.3688 (3)	0.5123 (2)	0.2014 (2)	0.0527 (7)	
C9	0.3518 (4)	0.5655 (2)	0.1084 (3)	0.0656 (9)	
H9	0.393805	0.556056	0.039524	0.079*	
C10	0.2713 (4)	0.6330 (2)	0.1194 (3)	0.0709 (10)	
H10	0.260753	0.669613	0.057878	0.085*	
C11	0.2056 (4)	0.6471 (2)	0.2215 (3)	0.0624 (8)	
H11	0.149448	0.691964	0.228923	0.075*	
C12	0.2277 (3)	0.59104 (19)	0.3115 (3)	0.0505 (7)	
C13	0.1742 (3)	0.58832 (18)	0.4275 (3)	0.0479 (6)	
C14	0.1634 (3)	0.53493 (17)	0.6065 (3)	0.0477 (6)	
C15	0.1941 (4)	0.48480 (19)	0.7055 (3)	0.0555 (7)	
H15	0.257444	0.441587	0.704734	0.067*	
C16	0.1273 (4)	0.5015 (2)	0.8048 (3)	0.0648 (8)	
H16	0.146543	0.469043	0.871969	0.078*	
C17	0.0323 (4)	0.5655 (2)	0.8065 (3)	0.0671 (9)	
H17	−0.010553	0.574915	0.874946	0.081*	
C18	−0.0003 (4)	0.6153 (2)	0.7104 (3)	0.0620 (8)	
H18	−0.064878	0.658034	0.711846	0.074*	
C19	0.0681 (3)	0.59909 (19)	0.6096 (3)	0.0525 (7)	

### Atomic displacement parameters (Å^2^)

**Table:**

	*U*^11^	*U*^22^	*U*^33^	*U*^12^	*U*^13^	*U*^23^
Ni1	0.0467 (3)	0.0507 (3)	0.0415 (3)	−0.0033 (2)	0.01543 (19)	0.0003 (2)
S1	0.0616 (4)	0.0555 (5)	0.0508 (4)	0.0004 (3)	0.0219 (3)	0.0050 (3)
S2	0.0559 (4)	0.0555 (5)	0.0600 (4)	0.0036 (3)	0.0168 (3)	−0.0024 (4)
O1	0.0971 (17)	0.0780 (17)	0.0525 (12)	0.0047 (14)	0.0353 (12)	−0.0035 (12)
N1	0.0491 (11)	0.0501 (14)	0.0458 (12)	−0.0054 (11)	0.0154 (9)	0.0003 (11)
N2	0.0453 (11)	0.0565 (15)	0.0430 (12)	−0.0044 (11)	0.0115 (9)	0.0005 (11)
N3	0.0428 (11)	0.0536 (14)	0.0452 (12)	−0.0062 (10)	0.0143 (9)	−0.0040 (11)
C1	0.0557 (15)	0.0431 (16)	0.0562 (16)	−0.0079 (13)	0.0158 (13)	−0.0041 (13)
C2	0.0726 (19)	0.0483 (18)	0.0645 (19)	−0.0051 (16)	0.0167 (16)	0.0015 (15)
C3	0.076 (2)	0.0449 (18)	0.086 (3)	0.0026 (16)	0.0166 (19)	−0.0030 (17)
C4	0.072 (2)	0.0515 (19)	0.084 (2)	0.0001 (16)	0.0237 (18)	−0.0175 (18)
C5	0.0619 (17)	0.0582 (19)	0.0615 (19)	−0.0075 (15)	0.0203 (14)	−0.0115 (15)
C6	0.0491 (14)	0.0458 (16)	0.0524 (15)	−0.0099 (12)	0.0137 (12)	−0.0088 (13)
C7	0.0550 (15)	0.0629 (19)	0.0441 (15)	−0.0077 (14)	0.0164 (12)	−0.0066 (14)
C8	0.0519 (14)	0.066 (2)	0.0414 (14)	−0.0036 (14)	0.0128 (11)	0.0009 (14)
C9	0.0668 (19)	0.086 (3)	0.0488 (17)	0.0057 (18)	0.0224 (14)	0.0096 (17)
C10	0.077 (2)	0.085 (3)	0.0524 (18)	0.011 (2)	0.0162 (15)	0.0239 (18)
C11	0.0569 (17)	0.068 (2)	0.0627 (19)	0.0094 (16)	0.0131 (14)	0.0150 (16)
C12	0.0437 (13)	0.0591 (18)	0.0496 (15)	0.0003 (13)	0.0117 (11)	0.0045 (14)
C13	0.0446 (13)	0.0515 (17)	0.0487 (15)	−0.0045 (13)	0.0114 (11)	−0.0023 (13)
C14	0.0468 (14)	0.0496 (16)	0.0486 (14)	−0.0112 (12)	0.0140 (11)	−0.0068 (12)
C15	0.0621 (16)	0.0563 (18)	0.0519 (16)	−0.0039 (15)	0.0205 (13)	−0.0047 (14)
C16	0.086 (2)	0.063 (2)	0.0501 (17)	−0.0095 (18)	0.0248 (16)	−0.0021 (15)
C17	0.079 (2)	0.072 (2)	0.0581 (19)	−0.0118 (18)	0.0330 (17)	−0.0155 (17)
C18	0.0654 (18)	0.063 (2)	0.0631 (19)	−0.0049 (16)	0.0261 (15)	−0.0161 (16)
C19	0.0494 (14)	0.0577 (18)	0.0529 (16)	−0.0101 (14)	0.0163 (12)	−0.0099 (14)

### Geometric parameters (Å, º)

**Table:**

Ni1—S1	2.1508 (9)	C5—H5	0.9300
Ni1—N1	1.871 (2)	C5—C6	1.409 (4)
Ni1—N2	1.843 (2)	C7—C8	1.496 (5)
Ni1—N3	1.952 (2)	C8—C9	1.380 (4)
S1—C1	1.761 (3)	C9—H9	0.9300
S2—C13	1.706 (3)	C9—C10	1.379 (5)
S2—C19	1.733 (3)	C10—H10	0.9300
O1—C7	1.229 (4)	C10—C11	1.396 (5)
N1—C6	1.405 (4)	C11—H11	0.9300
N1—C7	1.354 (4)	C11—C12	1.387 (4)
N2—C8	1.343 (3)	C12—C13	1.464 (4)
N2—C12	1.332 (4)	C14—C15	1.395 (4)
N3—C13	1.328 (4)	C14—C19	1.390 (4)
N3—C14	1.395 (3)	C15—H15	0.9300
C1—C2	1.387 (4)	C15—C16	1.381 (4)
C1—C6	1.408 (4)	C16—H16	0.9300
C2—H2	0.9300	C16—C17	1.384 (5)
C2—C3	1.387 (5)	C17—H17	0.9300
C3—H3	0.9300	C17—C18	1.368 (5)
C3—C4	1.382 (5)	C18—H18	0.9300
C4—H4	0.9300	C18—C19	1.403 (4)
C4—C5	1.380 (5)	Ni1—Ni1^i^	3.3305 (9)
			
N1—Ni1—S1	89.40 (8)	N1—C7—C8	111.2 (2)
N1—Ni1—N3	165.47 (10)	N2—C8—C7	111.5 (3)
N2—Ni1—S1	172.45 (8)	N2—C8—C9	120.0 (3)
N2—Ni1—N1	83.74 (11)	C9—C8—C7	128.6 (3)
N2—Ni1—N3	81.75 (10)	C8—C9—H9	120.6
N3—Ni1—S1	105.05 (8)	C10—C9—C8	118.8 (3)
C1—S1—Ni1	97.34 (10)	C10—C9—H9	120.6
C13—S2—C19	88.68 (14)	C9—C10—H10	119.5
C6—N1—Ni1	120.08 (18)	C9—C10—C11	121.0 (3)
C7—N1—Ni1	116.1 (2)	C11—C10—H10	119.5
C7—N1—C6	123.8 (2)	C10—C11—H11	121.5
C8—N2—Ni1	117.4 (2)	C12—C11—C10	116.9 (3)
C12—N2—Ni1	120.79 (19)	C12—C11—H11	121.5
C12—N2—C8	121.8 (3)	N2—C12—C11	121.4 (3)
C13—N3—Ni1	112.18 (18)	N2—C12—C13	108.4 (3)
C13—N3—C14	109.9 (2)	C11—C12—C13	130.2 (3)
C14—N3—Ni1	137.8 (2)	N3—C13—S2	116.7 (2)
C2—C1—S1	122.0 (2)	N3—C13—C12	116.8 (3)
C2—C1—C6	119.9 (3)	C12—C13—S2	126.4 (2)
C6—C1—S1	118.1 (2)	N3—C14—C15	126.3 (3)
C1—C2—H2	119.6	C19—C14—N3	113.5 (3)
C1—C2—C3	120.8 (3)	C19—C14—C15	120.1 (3)
C3—C2—H2	119.6	C14—C15—H15	121.1
C2—C3—H3	120.3	C16—C15—C14	117.8 (3)
C4—C3—C2	119.4 (3)	C16—C15—H15	121.1
C4—C3—H3	120.3	C15—C16—H16	119.3
C3—C4—H4	119.4	C15—C16—C17	121.5 (3)
C5—C4—C3	121.1 (3)	C17—C16—H16	119.3
C5—C4—H4	119.4	C16—C17—H17	119.0
C4—C5—H5	120.0	C18—C17—C16	122.0 (3)
C4—C5—C6	120.0 (3)	C18—C17—H17	119.0
C6—C5—H5	120.0	C17—C18—H18	121.5
N1—C6—C1	114.6 (2)	C17—C18—C19	116.9 (3)
N1—C6—C5	126.7 (3)	C19—C18—H18	121.5
C1—C6—C5	118.7 (3)	C14—C19—S2	111.0 (2)
O1—C7—N1	128.0 (3)	C14—C19—C18	121.7 (3)
O1—C7—C8	120.7 (3)	C18—C19—S2	127.2 (3)
			
Ni1—S1—C1—C2	173.1 (2)	C2—C1—C6—N1	−176.4 (3)
Ni1—S1—C1—C6	−6.3 (2)	C2—C1—C6—C5	1.4 (4)
Ni1—N1—C6—C1	3.2 (3)	C2—C3—C4—C5	0.7 (5)
Ni1—N1—C6—C5	−174.4 (2)	C3—C4—C5—C6	0.4 (5)
Ni1—N1—C7—O1	−176.1 (3)	C4—C5—C6—N1	176.0 (3)
Ni1—N1—C7—C8	3.1 (3)	C4—C5—C6—C1	−1.5 (4)
Ni1—N2—C8—C7	2.4 (3)	C6—N1—C7—O1	5.4 (5)
Ni1—N2—C8—C9	−177.8 (2)	C6—N1—C7—C8	−175.4 (2)
Ni1—N2—C12—C11	177.9 (2)	C6—C1—C2—C3	−0.3 (5)
Ni1—N2—C12—C13	−2.0 (3)	C7—N1—C6—C1	−178.3 (3)
Ni1—N3—C13—S2	174.38 (13)	C7—N1—C6—C5	4.1 (4)
Ni1—N3—C13—C12	−3.7 (3)	C7—C8—C9—C10	179.1 (3)
Ni1—N3—C14—C15	6.6 (5)	C8—N2—C12—C11	−2.0 (4)
Ni1—N3—C14—C19	−174.1 (2)	C8—N2—C12—C13	178.0 (2)
S1—Ni1—N1—C6	−6.08 (19)	C8—C9—C10—C11	−1.1 (5)
S1—Ni1—N1—C7	175.3 (2)	C9—C10—C11—C12	1.2 (5)
S1—C1—C2—C3	−179.6 (3)	C10—C11—C12—N2	0.3 (5)
S1—C1—C6—N1	2.9 (3)	C10—C11—C12—C13	−179.8 (3)
S1—C1—C6—C5	−179.2 (2)	C11—C12—C13—S2	5.9 (5)
O1—C7—C8—N2	175.8 (3)	C11—C12—C13—N3	−176.3 (3)
O1—C7—C8—C9	−4.0 (5)	C12—N2—C8—C7	−177.6 (2)
N1—Ni1—N2—C8	−0.6 (2)	C12—N2—C8—C9	2.2 (4)
N1—Ni1—N2—C12	179.4 (2)	C13—S2—C19—C14	−0.7 (2)
N1—C7—C8—N2	−3.5 (4)	C13—S2—C19—C18	178.1 (3)
N1—C7—C8—C9	176.7 (3)	C13—N3—C14—C15	−177.0 (3)
N2—Ni1—N1—C6	177.1 (2)	C13—N3—C14—C19	2.4 (3)
N2—Ni1—N1—C7	−1.6 (2)	C14—N3—C13—S2	−3.0 (3)
N2—C8—C9—C10	−0.6 (5)	C14—N3—C13—C12	178.9 (2)
N2—C12—C13—S2	−174.2 (2)	C14—C15—C16—C17	0.3 (5)
N2—C12—C13—N3	3.7 (4)	C15—C14—C19—S2	178.6 (2)
N3—Ni1—N1—C6	−179.9 (3)	C15—C14—C19—C18	−0.3 (4)
N3—Ni1—N1—C7	1.4 (5)	C15—C16—C17—C18	0.0 (5)
N3—Ni1—N2—C8	−179.9 (2)	C16—C17—C18—C19	−0.5 (5)
N3—Ni1—N2—C12	0.2 (2)	C17—C18—C19—S2	−178.1 (2)
N3—C14—C15—C16	179.1 (3)	C17—C18—C19—C14	0.7 (5)
N3—C14—C19—S2	−0.8 (3)	C19—S2—C13—N3	2.2 (2)
N3—C14—C19—C18	−179.7 (3)	C19—S2—C13—C12	−179.9 (3)
C1—C2—C3—C4	−0.8 (5)	C19—C14—C15—C16	−0.2 (4)

Symmetry code: (i) −*x*+1, −*y*+1, −*z*+1.

### Hydrogen-bond geometry (Å, º)

**Table:**

*D*—H···*A*	*D*—H	H···*A*	*D*···*A*	*D*—H···*A*
C9—H9···O1^ii^	0.93	2.27	3.135 (4)	155
C15—H15···S1	0.93	2.66	3.420 (3)	139

Symmetry code: (ii) −*x*+1, −*y*+1, −*z*.
